# The neurovanguard concept and real-world embracement

**DOI:** 10.1186/s13054-024-04941-z

**Published:** 2024-05-08

**Authors:** Fabio Silvio Taccone, Edith Elianna Rodriguez, Mario Zaccarelli, Elda Diletta Sterchele

**Affiliations:** Department of Intensive Care, Hôpital Universitaire de Bruxelles (HUB), Université Libre de Bruxelles (ULB), Route de Lennik, 808, 1070 Brussels, Belgium

Dear Editor,

We were delighted to receive the correspondence from Giglio et al. [1] regarding our recent viewpoint proposing the "NeuroVanguard" strategy for neuromonitoring in severe adult brain injury patients [2]. The authors aptly addressed potential challenges associated with implementing such an approach in real-world settings, particularly in low- and middle-income countries. These challenges include limited access to advanced invasive neuromonitoring modalities, such as intracranial pressure (ICP) monitoring, brain oxygenation assessment and metabolic monitoring. In these resource-constrained settings, reliance on clinical examination becomes paramount, along with the feasibility of assessing cerebral autoregulation at the bedside to guide therapeutic interventions, rather than solely focusing on brain compliance.

While we acknowledged the criticisms surrounding the use of ICP monitoring, particularly highlighted by the BEST-TRIP trial [3], several important points warrant attention. Firstly, it is noteworthy that less than 30% of patients in this trial exhibited elevated ICP. Consequently, the trial may not have been adequately identified the target population to demonstrate improved neurological outcomes with an ICP-guided therapy. Enhanced patient selection using non-invasive neuromonitoring modalities, such as cerebral ultrasound or automated pupillometry, could aid in screening individuals at high risk of intracranial hypertension. This targeted approach may justify the placement of an ICP monitor, even in resource-limited settings, where patients are most likely to benefit from ICP-guided therapy. Secondly, the BEST-TRIP trial revealed that patients undergoing ICP monitoring received less frequently therapies to reduce ICP, suggesting an overestimation or inaccuracy of clinical examination and brain imaging in identifying situations requiring such interventions. Lastly, in this study, there was a non-significant 5% increase in the proportion of patients with favorable outcomes in the ICP group; this raises the possibility that the study may have been underpowered to detect clinically relevant differences between the intervention arms.

We concur with the authors' perspective on the paramount importance of clinical examination as the primary neuromonitoring tool, underscoring its significance when conducted not only by physicians but notably by nurses. However, performing an adequate neurological examination in critically ill patients by non-neurologists may face several limitations, such as lack of expertise (e.g. leading to potential errors in assessment and interpretation), complexity of findings, presence of subtle symptoms (e.g. apraxia, dysgraphia), the presence of confounders (e.g. in particular the use of sedatives or intubation), and the lack of specialized equipments for some aspects of the neurological examination, such as cranial nerve function or reflexes. Moreover, most of non-neurologist healthcare providers present the so-called “neurophobia”, which refers to the fear or apprehension surrounding neurological examination and brain diseases due to the limited knowledge in this field [4]. Simplifying the neurological examination with specific scales like the Glasgow Coma Scale can pose challenges in accurately assessing neurological deterioration and determining appropriate therapeutic interventions in this context.

Finally, we respectfully disagree with the authors regarding the significance of cerebral autoregulation in guiding therapeutic interventions in the field of acute brain injured patients. While it has been suggested to elevate mean arterial pressure to potentially mitigate ICP surge in severe traumatic brain injury (TBI) patients, based on the efficacy of cerebral vasculature adjustment to pressure stimuli, the assessment of dynamic autoregulation poses challenges due to the variability in available indices and the requirement for specialized software and expertise [5], which may not be readily accessible in low- and middle-income countries. Furthermore, the intricate dynamics of cerebral autoregulation are influenced by factors such as metabolic activity, microcirculatory hematocrit, and perivascular pH, which cannot be adequately captured solely through pressure-autoregulation assessments. Consequently, while cerebral autoregulation monitoring shows promise in optimizing cerebral perfusion in TBI patients, its clinical applicability and feasibility require further validation across diverse patient populations and beyond specialized centers with extensive experience in neurocritical care.

## Data Availability

Not applicable. All cited articles are available in the respective journals.

## Abbreviations

ICP: Intracranial pressure
TBI: Traumatic brain injury

## References

[1] Giglio A, Pino M, Ferre A, Reccius A (2024). Adapting NeuroVanguard to real-world challenges. Crit Care.

[2] Rodriguez EE, Zaccarelli M, Sterchele ED, Taccone FS (2024). "NeuroVanguard": a contemporary strategy in neuromonitoring for severe adult brain injury patients. Crit Care.

[3] Chesnut RM, Temkin N, Carney N, Dikmen S, Rondina C, Videtta W, Petroni G, Lujan S, Pridgeon J, Barber J, Machamer J, Chaddock K, Celix JM, Cherner M, Hendrix T, Global Neurotrauma Research Group (2012). A trial of intracranial-pressure monitoring in traumatic brain injury. N Engl J Med.

[4] Jozefowicz RF (1994). Neurophobia: the fear of neurology among medical students. Arch Neurol.

[5] Burma JS, Roy MA, Kennedy CM, Labrecque L, Brassard P, Smirl JD. A systematic review, meta-analysis, and meta-regression amalgamating the driven approaches used to quantify dynamic cerebral autoregulation. J Cereb Blood Flow Metab. 2024: 271678X241235878 10.1177/0271678X241235878 PMC11342731 38635887

